# Long QTc Syndrome Type 2 Presenting in a Postpartum Patient on Medroxyprogesterone

**DOI:** 10.1155/2014/676080

**Published:** 2014-10-21

**Authors:** John Kern, Margaret Duffy, Corinne Kern, Victor Mazza

**Affiliations:** ^1^Internal Medicine Residency Program, Rutgers New Jersey Medical School, Rutgers University, 150 Bergen Street, UH-I248, Newark, NJ 07101, USA; ^2^Hackensack University Medical Center, 30 Prospect Avenue, Hackensack, NJ 07601, USA

## Abstract

*Introduction*. Congenital long QT syndrome type 2 (LQTS2) is a rare inherited cardiac abnormality resulting in increased risk of polymorphic ventricular tachycardia (PVT). *Case Description*. A 21-year-old postpartum female presented with syncopal episode after phone alarm. She was noted to have PVT on telemetry monitoring in the emergency department. EKG revealed QTc of 530. The patient's only medication was medroxyprogesterone. She ultimately received a dual chamber pacemaker with ICD. *Discussion*. LQTS2 is associated with alarm sounds as a precipitating factor. Postpartum hormonal shifts as well as medroxyprogesterone have significant effect on native QTc duration.

## 1. Introduction

Congenital long QT syndrome type 2 (LQTS2) is a rare inherited cardiac abnormality resulting in QT prolongation associated with the risk of polymorphic ventricular tachycardia. This patient's risk of having a cardiac event was increased due to her postpartum state.

## 2. Case Description

A 21-year-old, two-month postpartum female presented following a three-day history of syncopal episodes. The first occurred when her son set off her cell phone alarm and the second with her son playing a loud video game. She was placed on telemetry monitoring in the emergency department just prior to a “syncopal” episode and was found to be in polymorphic ventricular tachycardia (PVT). EKG revealed a QTc of 530 msec. The patient was also found to be hypokalemic. Her QT interval remained prolonged following repletion. The patient's only medication was medroxyprogesterone acetate. She has no family history of sudden cardiac death. She failed therapy with beta blockade and on the fourth day of admission a dual chamber pacemaker with ICD was placed for secondary prevention of sudden cardiac death. She had no further telemetry events and was discharged home to follow up with outpatient electrophysiology.

## 3. Discussion

LQTS2 is associated with alarm sounds as a precipitating factor, as it was in this case. The hormonal impacts have been recently described. Estrogen results in QTc prolongation while progesterone results in QTc shortening [1]. The dramatic shifts in estrogen/progesterone levels in the postpartum state have resulted in the initial cardiac presentation of LQTS2 in several patients. While endogenous progesterone provides protection via activation of endothelial nitrogen oxide synthetase, synthetic medroxyprogesterone acetate does not have this protective mechanism [2]. Therefore it could be implied that the use of medroxyprogesterone acetate could precipitate arrhythmias in a patient predisposed to such events. LQTS2 is a rare syndrome that should be considered in the differential when assessing postpartum patients with complaints of syncope.

LQTS2 exacerbation postpartum has been well described in literature. There have been recent implications of the potential exacerbation of LQTS2 by the use of medroxyprogesterone acetate. The hormonal effects on QT length have been described in both animal and human models. Hormonal effects in females as they reach puberty were shown in a study by Locati et al., which highlighted the QTc changes in women from age 15 to 50 and correlated with a 10–20 ms shift in the average QTc, thought secondary to increased estrogen production [1]. In a large retrospective study by Rashba et al. the postpartum risk of cardiac events was increased specifically in LQTS2 [3]. Nakamura et al. highlighted the role of progesterone in shortening QT interval and its protective nature regarding the prevention of cardiac arrhythmias in this context [4]. Additionally progesterone levels, which are increased during pregnancy, resulted in a decrease in arrhythmogenic potential. The mechanism of progesterone is related to the activation of endothelial nitric oxide synthetase. While medroxyprogesterone acetate functions in a manner similar to endogenous progesterone it does not activate endothelial nitric oxide synthetase, thus conferring no cardiac protection or QT shortening [2]. In our patient this lack of peripartum cardiac protection increased her risk of experiencing a cardiac event.

Females with LQTS2 are at increased risk of developing arrhythmias when compared to males of the same age group. In a risk analysis it was shown that patients with LQTS2 and QTc greater than 498 msec carry with them a relative risk of a cardiac event by age 40 of 8.36. Furthermore there is a 50% likelihood of experiencing a cardiac event before age 40 in the absence of treatment [5]. This case highlights the importance of evaluation of syncope in the postpartum patient and the careful consideration of contraception choice in patients with LQTS2.
